# Combination of anti-vascular agent - DMXAA and HIF-1α inhibitor - digoxin inhibits the growth of melanoma tumors

**DOI:** 10.1038/s41598-018-25688-y

**Published:** 2018-05-09

**Authors:** Ryszard Smolarczyk, Tomasz Cichoń, Ewelina Pilny, Magdalena Jarosz-Biej, Aleksandra Poczkaj, Natalia Kułach, Stanisław Szala

**Affiliations:** 1 Center for Translational Research and Molecular Biology of Cancer Maria Sklodowska-Curie Institute - Oncology Center, Gliwice Branch, Wybrzeże Armii Krajowej Street 15, 44-101 Gliwice, Poland; 2 Department of Organic Chemistry, Biochemistry and Biotechnology, Silesian University of Technology, Gliwice, Poland; 3 Department of Animal Physiology and Ecotoxicology, University of Silesia, Bankowa 9 St., Katowice, Poland

## Abstract

Vascular disrupting agents as DMXAA inhibit tumor growth only for a short period of time followed by rapid tumor regrowth. Among others, hypoxia and presence of transcription factor HIF-1α are responsible for tumors regrowth. The aim of our study was to investigate the inhibition of murine melanoma growth by combining two agents: anti-vascular - DMXAA and the HIF-1α inhibitor - digoxin and explaining the mechanism of action of this combination. After DMXAA treatment tumor size was reduced only for a limited time. After 7 days regrowth of tumors was observed and number of vessels was increased especially in tumor’s peripheral areas. DMXAA also induced an influx of immune cells: macrophages, CD8+ cytotoxic lymphocytes, NK cells, CD4+ lymphocytes. Administration of digoxin alone inhibited the growth of tumors. Administration of both agents in the proper sequence significantly inhibited the regrowth of tumors better than either agents alone. Combination therapy reduced number of newly formed vessels. In tumors of mice treated with combination therapy, the number of macrophages M1, CD8+ cytotoxic lymphocytes, NK cells and to a lesser extent CD4+ cells was increased. The combination of anti-vascular agents with HIF-1α inhibitors appears to be an effective therapeutic option.

## Introduction

Targeting of tumor associated blood vessels is one of the goals of anti-cancer therapy. Currently, two therapeutic strategies are known: one of them is anti-angiogenic therapy, which inhibits the formation of new blood vessels, the second one, anti-vascular therapy, destroys existing blood vessels in tumors. A significant limitation of anti-angiogenic therapy is drug resistance emergence. Anti-vascular drugs (Vascular Disruptive Agents – VDA) specifically destroy existing blood vessels in tumor and reduce the tumor volume^1^. Around the damaged blood vessels, extensive areas of hypoxia and necrosis appear. Enhanced infiltration of immune cells is also observed. The most known anti-vascular drugs include DMXAA, combretastatin A-4 disodium phosphate (CA4P), Plinabulin (NPI-2358). CA4P and NPI-2358 are microtubule destabilizing drugs^2,3^. DMXAA (5,6-Dimethylxanthenone-4-acetic Acid; also known as: ASA404, Vadimezan) is a xanthene which induces apoptosis in tumor vascular endothelium cells what results in necrosis appearance at tumor core. It activates the TANK-binding kinase 1/interferon regulatory factor 3 (TBK1/IRF3) signaling pathway in leukocytes, inducing type-I-interferon (IFN-I) production^4,5^. DMXAA vascular disrupting properties are partly mediated by TNF-α signaling^6^. DMXAA activates the mitochondria- and endoplasmic reticulum-associated protein known as stimulator of interferon genes (STING)^7,8^.

Promising results of DMXAA obtained in preclinical studies on mice have not been confirmed in research involving humans. The reason for the lack of efficacy of this therapeutical approach is the specificity of only murine STING protein stimulation by DMXAA^9,10^. Currently the compounds interacting with a human STING protein such as synthetic cyclic dinucleotide (CDN) - cyclic guanosine monophosphate-adenosine monophosphate (cyclic GMP-AMP, or cGAMP) are known^7,11,12^. cGAMP activate STING pathway, through bounding to STING protein, followed by phosporylation of TANK-binding kinase 1 (TBK-1) and Interferon Regulatory Factor 3 (IRF-3) induce production of interferon-β^13,14^. Other compounds are derivatives of DMXAA^15,16^, that activate human STING protein as effectively as DMXAA does in mice.

However, the effect of anti-vascular drugs has its limitations. Destruction of neoplastic blood vessels is associated with the appearance of inflammation, hypoxia and activation of HIF-1α protein in tumors, which in turn leads to formation of new blood vessels and tumor regrowth^17–19^. Digoxin is an inhibitor of HIF-1α protein translation and HIF-2α mRNA expression^17,20^. Digoxin reduces the amount of HIF-1α transcription factor, and consequently inhibits the growth of tumors in mice^20^. Recent data also indicate that digoxin inhibits endothelial focal adhesion kinase and angiogenesis^21^. The aim of our work was to combine the action of an anti-vascular drug - DMXAA with HIF-1α inhibitor - digoxin in the treatment of mice with B16-F10 melanoma tumors and to examine the mechanism of action of this combination.

## Results

### The combination of DMXAA and digoxin inhibits the growth of B16-F10 murine melanoma

**Figure 1:**
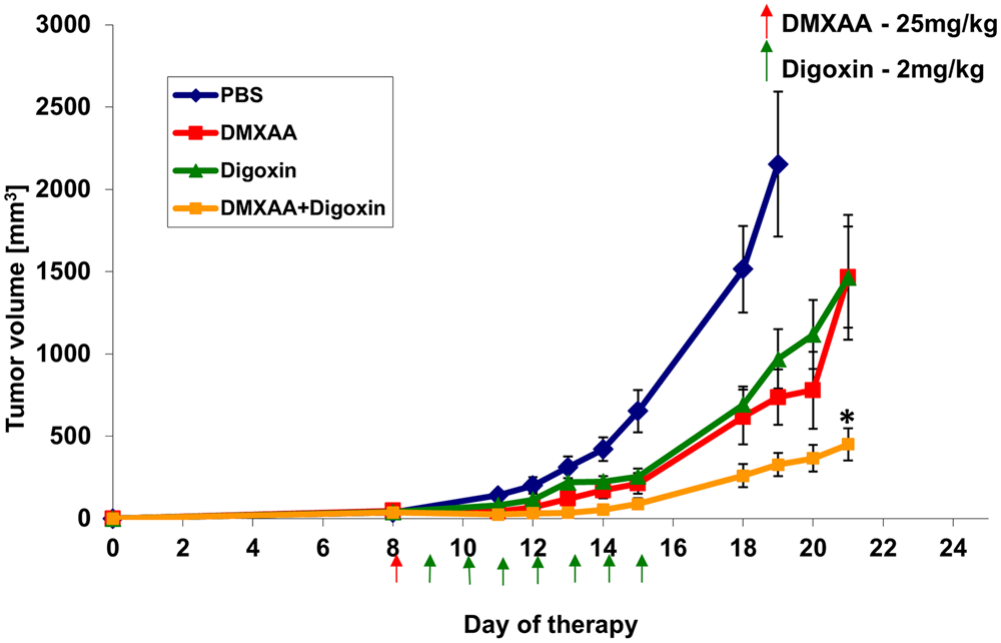
Treatment of mice bearing B16-F10 tumors with DMXAA and digoxin. DMXAA was administered once (25 mg/kg) and digoxin 7 times (2 mg/kg). Inhibition of tumor growth was statistically significant (*p < 0,05) after administration of combined therapy.

Single, intraperitoneal administration of DMXAA at a dose of 25 mg/kg body weight inhibits tumor growth in treated mice compared to control mice that received a PBS^−^ solution (Fig. 1). However, since 4^th^ day after administration tumor regrowth was observed. Intraperitoneal administration of digoxin alone (7 times) at a dose of 2 mg/kg body weight inhibits the growth of melanoma tumors in mice. Combination of DMXAA and digoxin inhibits tumor progression in treated mice more effectively than either of the compounds alone. At the 19^th^ day of the experiment the volume of tumors in mice treated with DMXAA was about 65% smaller than the volume of control tumors. In digoxin-treated mice, the tumor volume was 54% smaller than control tumors. Whereas volume of tumors in mice treated with the combination therapy was 84% smaller than in control mice. Inhibition of tumor growth after administration of DMXAA and digoxin was statistically significant compared to each of the compounds alone (p < 0,005).

### Administration of DMXAA and digoxin reduces the number of blood vessels in the tumor and increases the infiltration of immune cells

**Figure 2:**
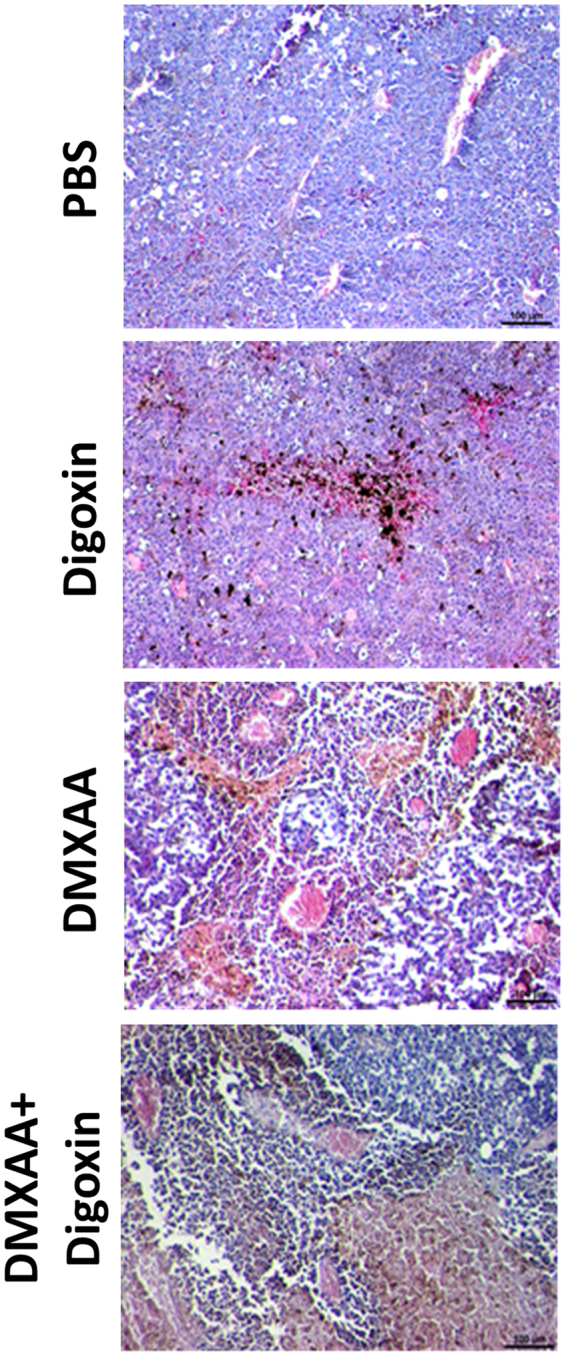
Hematoxylin and eosin staining after administration of DMXAA and digoxin. After administration of DMXAA and combined therapy destroyed blood vessels and infiltration of immune cells in the tumors were observed. Digoxin was shown to reduce the area of necrosis in tumors. Lens magnification 10×.

**Figure 3:**
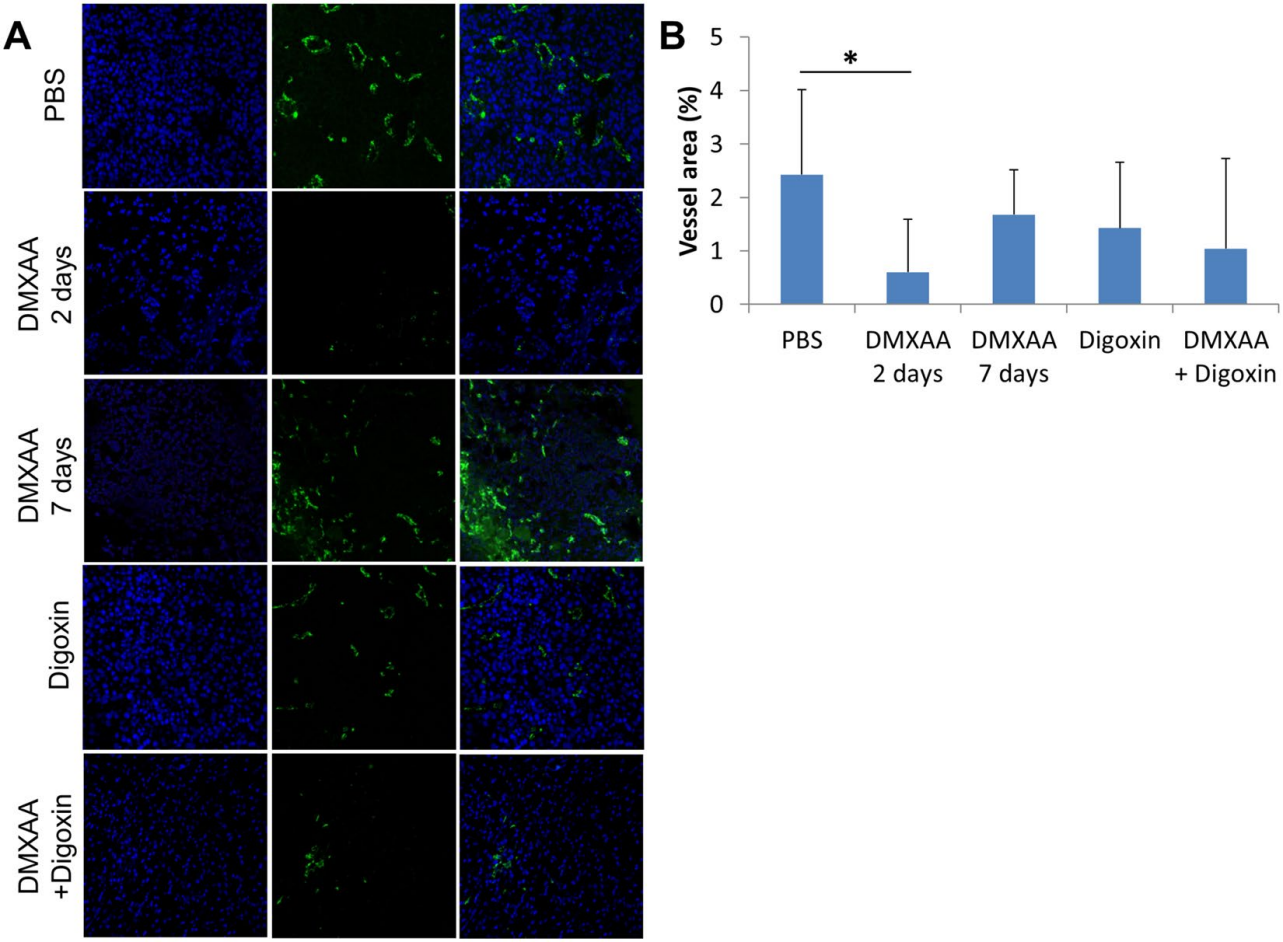
The area occupied by blood vessels in tumors after treatment with DMXAA and digoxin. DMXAA significantly decreases the area of blood vessels in the tumor (*p < 0,05). After digoxin administration it is only slightly reduced. 7 days after DMXAA administration the vessels area increases while in the group treated with the combined therapy it is significantly smaller.

Damaged blood vessels in tumors and significant infiltration of immune cells were observed after two days after DMAA was administered to mice with tumors (vol. about 40 mm) (Figs 2 and 3A,B). The area occupied by blood vessels within the tumor was reduced from 2.4% in the control tumors to 0.6% in the group that received DMXAA. However, after 6 days, regrowth of tumors in peripheral zone was observed, and area occupied by the blood vessels increased to 1.7%. Administration of digoxin alone (5 times) also reduced the area occupied by blood vessels on 6^th^ day of the therapy from 2.4% in the group receiving PBS^−^ up to 1.4% in treated group. Administration of both DMXAA and digoxin (after 6 days of therapy) significantly reduces the area occupied by blood vessels to 1%. In addition, significant infiltration of immune cells was observed around the damaged blood vessels, in group that received DMXAA alone and in group that received DMXAA and digoxin (Fig. 2).

### HIF-1α identification in melanoma tumors

**Figure 4:**
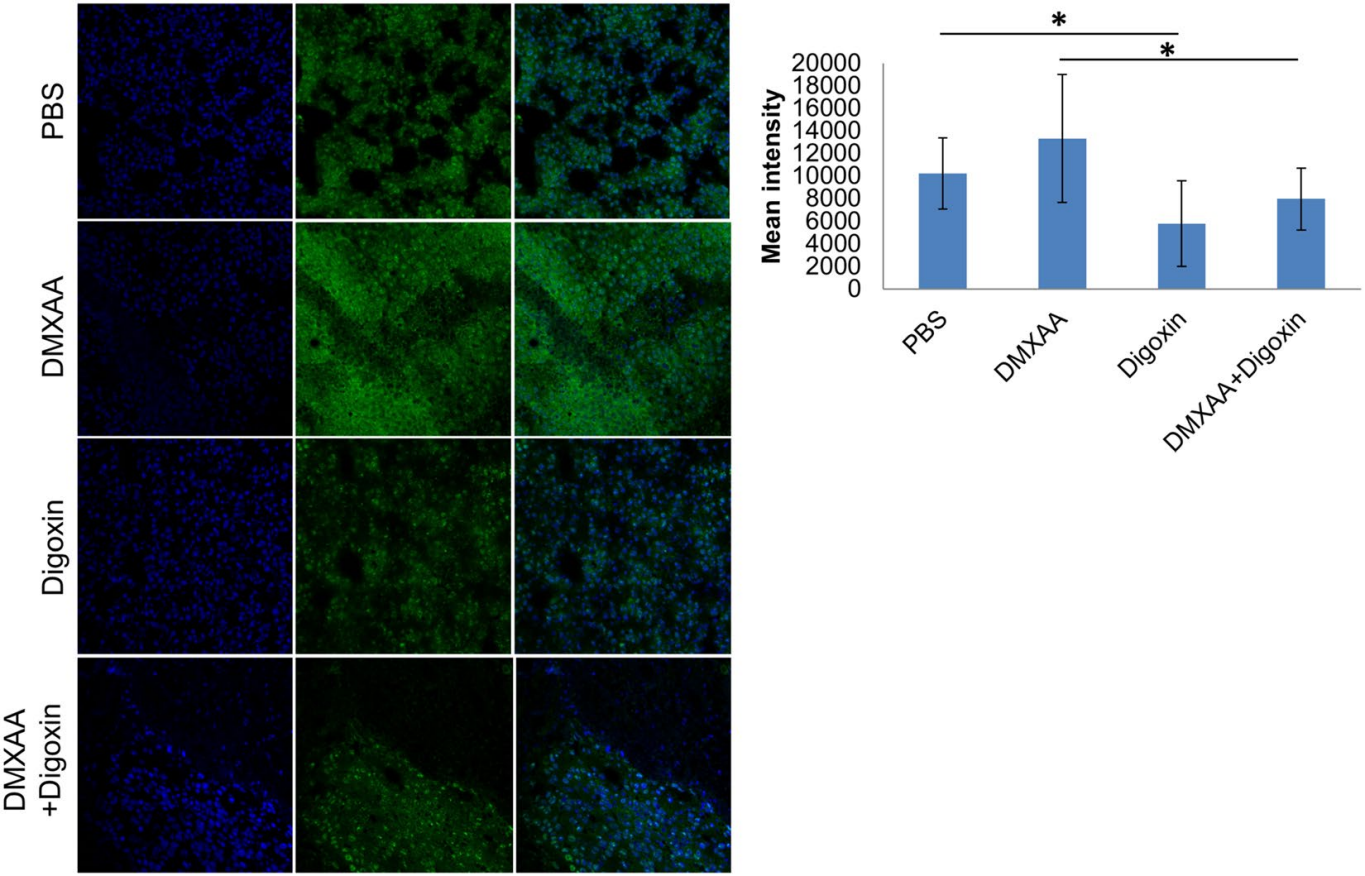
HIF-1α identification in tumors. The amount of HIF-1α factor decreased in tumors of mice that received digoxin and combined therapy compared to mice that received PBS and DMXAA (p < 0.05). In the group of mice that received DMXAA, the amount of HIF-1α increased (no statistical significance).

Administration of DMXAA to mice bearing B16-F10 melanoma increases the amount of HIF-1α transcription factor in tumors comparing to the control mice that received the PBS^−^ solution. Administration of digoxin to mice, both the group that received digoxin alone and the group that previously received DMXAA, reduced the amount of HIF-1α transcription factor in tumors – the intensity of the green fluorescence after antibody against HIF-1α staining was lower compared to the mice that received PBS^−^ and DMXAA (Fig. 4A,B).

### The effect of DMXAA and digoxin on M1 macrophages infiltration in tumors

**Figure 5:**
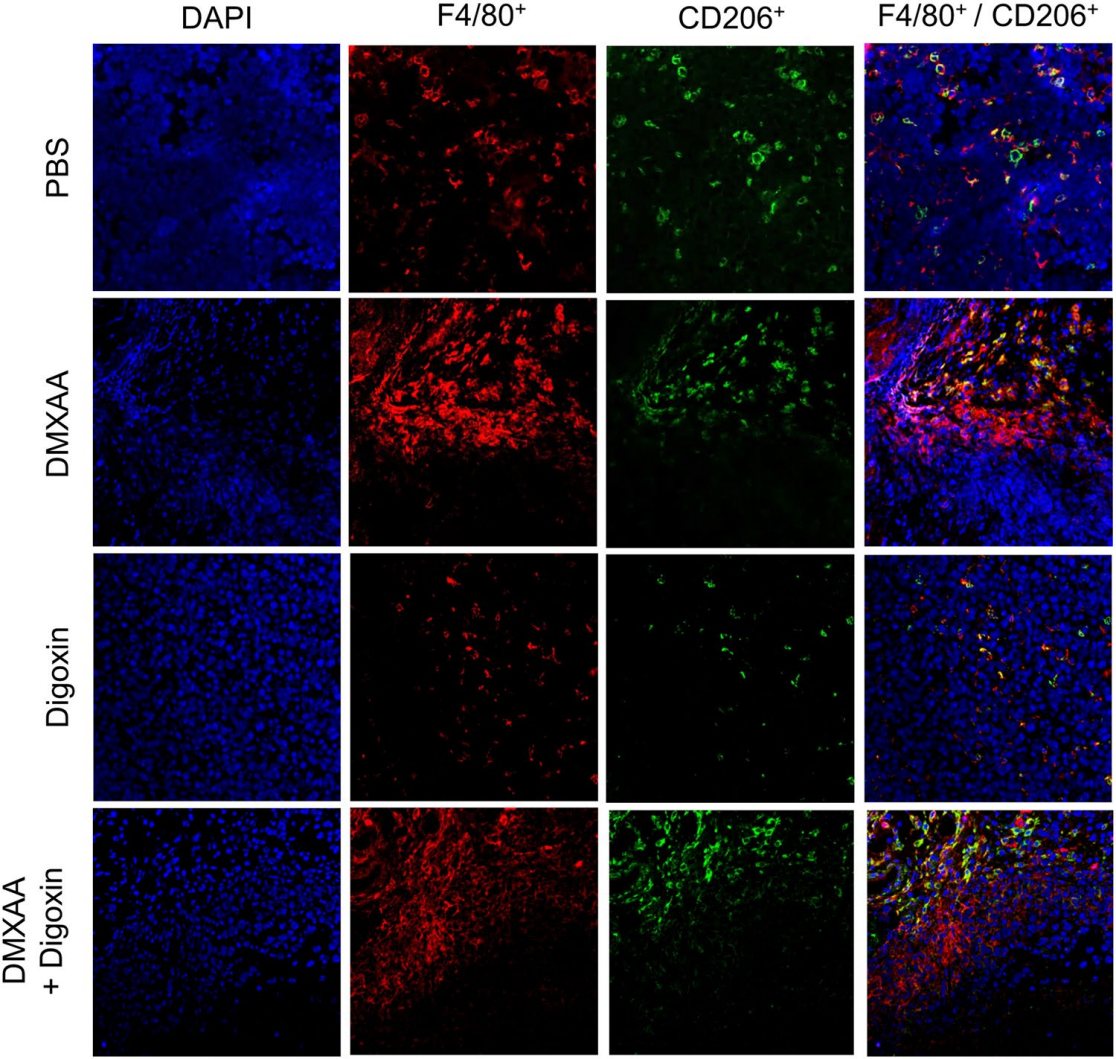
Identification of macrophages in murine melanoma after administration of DMXAA and digoxin. Cytotoxic (M1) macrophages F4/80^+^/CD206^−^ infiltration was observed after administration of DMXAA and digoxin. Digoxin alone does not alter the level of macrophages F4/80^+^/CD206^+^ (M2) and F4/80^+^/CD206^−^ (M1).

Administration of both DMXAA alone and the combination of DMXAA and digoxin caused cytotoxic M1 macrophages (F4/80^+^/CD206^−^) infiltration. The administration of digoxin alone does not change the number of M2 (F4/80^+^/CD206^+^) and M1 (F4/80^+^/CD206^−^) macrophages. In mice treated with DMXAA alone as well as with DMXAA and digoxin combination, M1 macrophages infiltration was observed in areas where tumor cells were present, followed by migration of M2 macrophages repairing the lesion site (Fig. 5).

### Identification of CD4^+^, CD8^+^ and NK immune cells in melanoma tumors

**Figure 6:**
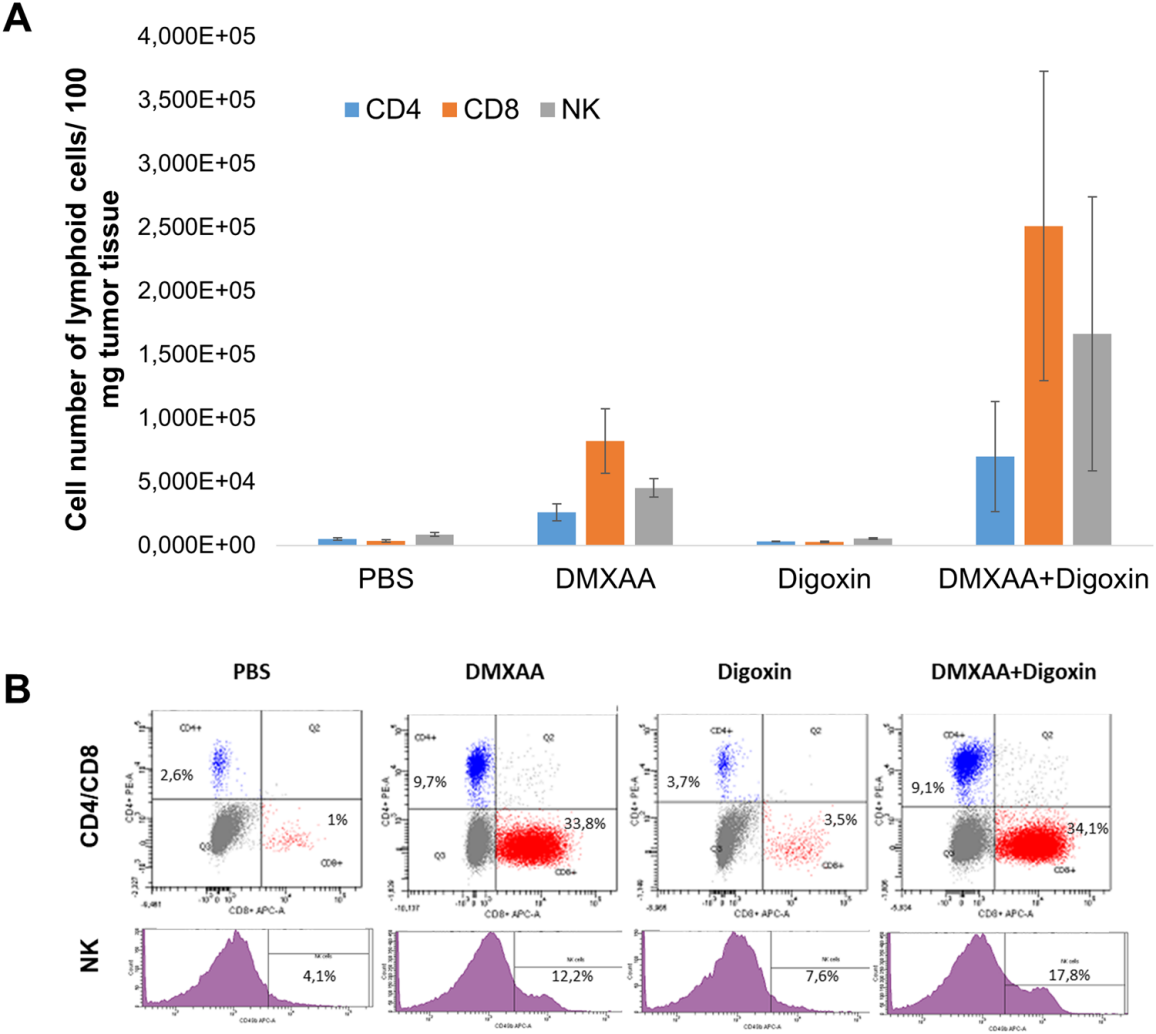
Presence of immune cells in murine melanoma after administration of DMXAA and digoxin. DMXAA alone and the combination of DMXAA with digoxin increase the levels of CD8^+^ lymphocytes, NK cells and to a lesser extent CD4^+^ lymphocytes (**A**). Digoxin alone does not change CD8^+^, CD4^+^, NK cells contribution comparing to control (PBS^−^). Representative plots of cytometric analysis (**B**).

Administration of both DMXAA alone and a combination of DMXAA and digoxin to mice bearing melanoma significantly increased the percentages of CD8^+^ and NK cells. Percentage of CD8^+^ cells increased from 1.4% in the group that received PBS^−^ solution to 26.1% in the group receiving DMXAA and to 32.3% in the group that received the combination of DMXAA and digoxin. The level of CD4^+^ cells was also changed from 2.4% - (PBS^−^) to 8.7% (DMXAA) and 7.5% (DMXAA + digoxin). Percentages of both CD8^+^, CD4^+^ and NK cells did not significantly change in the group that received digoxin alone in relation to the group receiving PBS^−^ (Fig. 6A,B).

### A proposed scheme of action of DMXAA and digoxin

**Figure 7:**
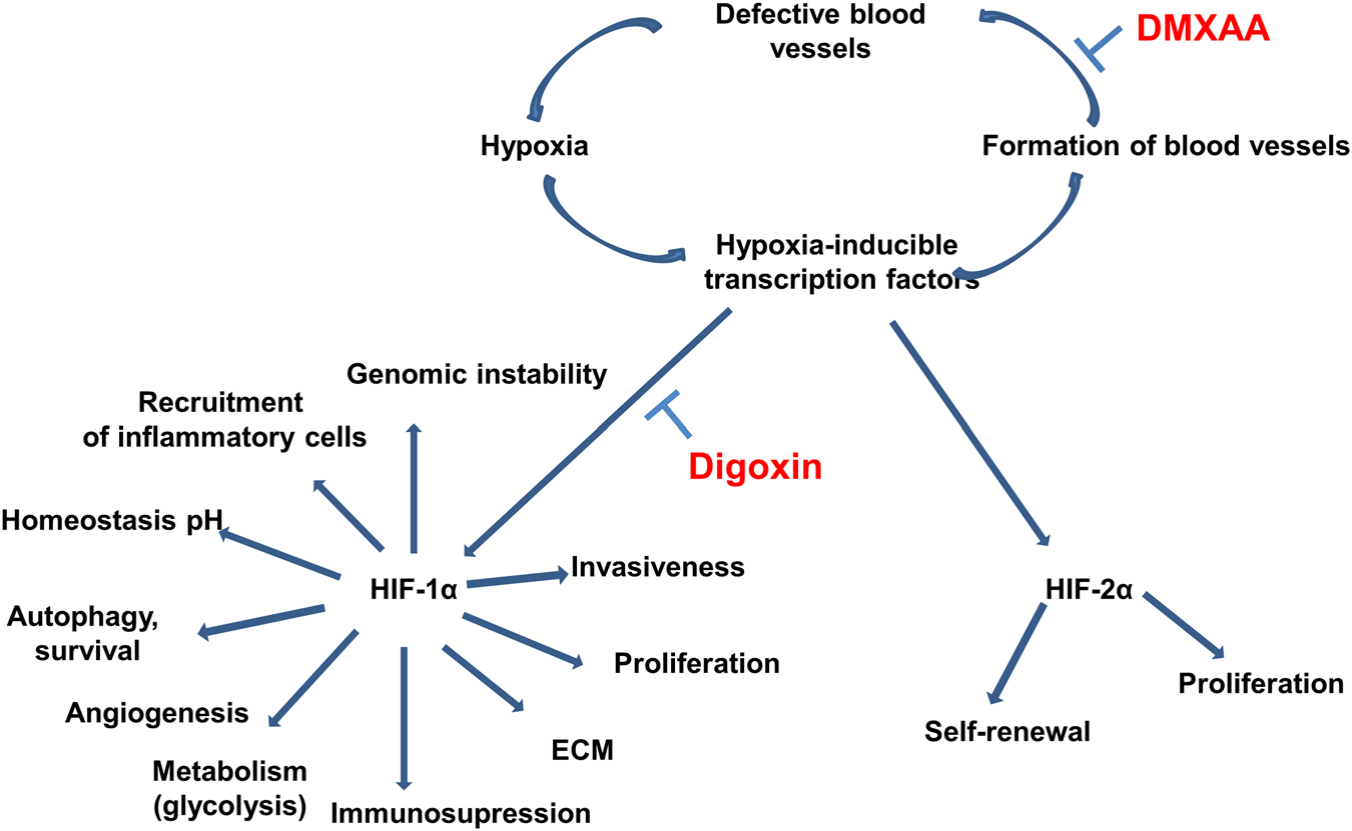
Scheme of action of the drugs. DMXAA administration to mice bearing tumors leads to the destruction of existing tumor blood vessels. Destruction of the vessels results in the formation of hypoxia areas where HIF-1α transcription factor expression is increased. Activation of HIF-1α triggers several mechanisms that promote tumor growth. Inhibition of HIF-1α after digoxin administration supresses tumor regrowth.

A single intraperitoneal administration of DMXAA causes destruction of existing blood vessels in tumor. Destruction of blood vessels causes the formation of hypoxia areas in which the level of the HIF-1α transcription factor increases. One of the effects of increased amount of HIF-1α is the formation of new blood vessels, which results in tumor regrowth. In addition, activation of HIF-1α activates a number of mechanisms that promote tumor growth, such as immunosuppression, genetic instability, activation of autophagy, increased invasiveness and cancer cell survival. Inhibition of HIF-1α activity after digoxin administration inhibits the regrowth of tumors (Fig. 7).

## Discussion

Therapy that targets blood vessels effectively inhibits the growth of tumors. The anti-angiogenic therapy, which inhibits formation of new capillaries, has its limitations involving the possible resistance to anti-angiogenic drugs. Activation of alternative mechanisms of new blood vessels formation is stimulated. The use of anti-vascular drugs that destroy existing blood vessels in tumors seems to be effective therapeutical approach. These drugs specifically recognize and destroy blood vessels in tumors. There are some antivascular drugs that are used in early-phase of clinical trials. CKD-516 is a newly developed vascular disrupting agent. Study confirms the efficacy of this drug in patients with advanced solid tumors^22^. 5,6-Dimethylxanthenone-4-acetic Acid (DMXAA, Vadimezan, ASA404) appears to be the most thoroughly tested drug in this group. The drug is effective in inhibiting the growth of murine glioma, acute myeloid leukaemia, B16.F10 melanoma, endocrine tumors, non-small cell lung cancer^6–8,12,23^. Our results confirm the effectiveness of inhibition of B16-F10 murine melanoma tumors growth after the administration of DMXAA in a dose of 25 mg/kg body weight.

However, clinical studies with DMXAA conducted on patients did not confirm the expected results. The reason for the failure were differences in structure of murine and human stimulator of the interferon genes protein (STING)^9,10^. Nowadays the intensive research efforts are made for the human analogue of this drug. Downey’s *et al*. and Corrales’ *et al*. results indicate that synthetic cyclic dinucleotides activate STING protein in humans as DMXAA does in mice^7,12^. Synthetic cyclic dinucleotides (CDNs) are cyclic guanosine monophosphate-adenosine monophosphate (cyclic GMP-AMP, or cGAMP)^7,11,12^. cGAMP binds to STING, leading to the activation of IRF3 and induction of interferon-I cytokines^13,14^. Other studies indicate that the modification of the DMXAA structure causes that the compound becomes ligand of human STING protein^15,16^. Thus, the analogues of the DMXAA compound effectively stimulate the STING receptor (work in humans like DMXAA does in mice) which is no longer a species barrier.

The problem with the use of anti-vascular drugs is the stimulation of necrosis around the damaged blood vessels and an increase of hypoxia areas in tumor that leads to HIF-1α transcription factor activation. The factor is responsible for a number of processes in tumors. Among others, it stimulates the formation of new blood vessels, increases survival, proliferation potential and invasiveness of tumor cells and finally activates immunosuppression^17–19,24^.

In our study, 7 days after the administration of DMXAA, an upregulation of HIF-1α level and the number of blood vessels in the tumor were observed what in consequence led to tumor regrowth. Tumors regrowth peripherally with central area occupied by extensive necrosis and with increased number of blood vessels in marginal zone. Therefore, it seems that downregulation of the HIF-1α transcription factor may bring an additional therapeutic effect in approach using anti-vascular drugs. Many inhibitors of Hypoxia Inducible Factor 1 are currently known^25^. One of them is a drug used in treatment of cardiovascular system diseases - digoxin. The work of Zhang H and co-workers has shown that digoxin reduces the expression of HIF-1α in cells^20^. Other work showed that the density of blood vessels in prostate cancer was inhibited after digoxin treatment^26^. Our studies confirm these observations and show that the use of digoxin in the treatment of murine B16-F10 melanoma at a dose of 2 mg/kg body weight: reduces level of HIF-1α in tumors of treated mice (the factor is not inhibited but its quantity is significantly reduced), inhibits the growth of tumors, and also, in combination with anti-vascular agent DMXAA, restrains tumor regrowth (compared with mice that received only DMXAA). We observed a reduction of blood vessels area after digoxin treatment but it wasn’t statistically significant. It seems that the use of an agent strongly inhibiting HIF-1α could bring even better therapeutic effect.

DMXAA, besides the destruction of existing vessels, stimulates the immune response in mice. The stimulation is carried out by reprogramming proangiogenic and immunosuppressive M2 macrophages towards cytotoxic M1 phenotype^12,27^. Hypoxia, emerged in tumors, causes suppression of T lymphocytes, inhibits maturation of dendritic cells and activation of NK cells. Treg cells, tumor-associated macrophages and neutrophils are activated^28^. Our studies have shown that DMXAA increases the levels of M1 macrophages in tumors of both groups – the one that received DMXAA alone and in the group that received DMXAA and digoxin combination. We also observed that cytotoxic M1 macrophages that destroyed neoplastic cells were followed by M2 macrophages repairing damaged tumor sites. Besides the stimulation of macrophages to destroy cancer cells, we observed a significant increase in the percentages of CD8^+^ cytotoxic lymphocytes and NK cells and to a lesser extent increased percentage of CD4^+^ cells in the groups that received both DMXAA alone and the combination of DMXAA and digoxin. Digoxin alone did not induce significant changes in the number of immune cells compared to the control group.

The improvement in the therapeutic efficacy of DMXAA in combination with digoxin is therefore both the effect of inhibiting the HIF-1α protein and stimulating the immune system that activates macrophages, CD8^+^ cytotoxic lymphocytes, NK cells and CD4^+^ lymphocytes to destroy cancer cells in the tumor^29^.

The increase in the number of blood vessels is associated with a rapid regrowth of the tumor. In combined therapy, the number of vessels does not increase as with monotherapy with DMXAA. Using a combination of both therapeutic agents the growth of tumors may be controlled.

The use of anti-vascular drugs as DMXAA in combination with HIF-1α transcription factor inhibitors may supress tumor regrowth.

## Methods

### Mice and cell line

Mice (6- to 8-week-old, C57Bl/6NCrl females) were obtained from Charles River Breeding Laboratories. Mice were housed in a pathogen-free facility in SPF standard. Animals were treated in accordance with the European Community guidelines. The protocol was approved by the Committee on the Ethics of Animal Experiments of the Local Ethics Commission (Medical University of Silesia, Katowice, Poland). Murine melanoma B16-F10 (ATCC) were propagated in RPMI 1640 supplemented with 10% fetal bovine serum. Cell cultures were maintained in a standard 37 °C/5% CO_2_ incubator. Cells were passaged every 3–4 days.

### Agents used in the experiments

DMXAA was purchased from Slleckchem (Houston, TX, USA), catalog number: S1537. Digoxin was purchased from Sigma-Aldrich (St Louis, MO, USA), catalog number: D6003.

### Therapy of mice bearing B16-F10 melanoma tumors

Seven days after inoculating mice (lower flank) with B16-F10 melanoma cells (2 × 10^5^ cells/100 µL PBS-), intraperitoneal injection of DMXAA-treated mice were initiated at dose 25 mg/kg body weight. Next day after DMXAA injection digoxin (2 mg/kg body weight) was administered intraperitoneally to appropriate groups of mice (digoxin and DMXAA + digoxin groups). Digoxin was administered daily for seven consecutive days. Tumor volume was monitored. Tumors were measured with calipers and tumor volumes were determined using the formula: volume = width 2 × length × 0.52.

### Immunohistochemistry

In days 2 and 7 after DMXAA and digoxin administrations tumors were embedded in liquid nitrogen and sectioned into 5μm slices. To determine the presence of the blood vessels in collected tumors, frozen sections were stained using antibody directed against CD31 antigen (Abcam; Ab7388, 1:50, Cambridge, UK). Area occupied by blood vessels was counted with ImageJ software (NIH). Stained blood vessels were counted in 5 randomly chosen fields (magn. 20×) per section in 4 tumors of each group. To determine the presence of macrophages with M2 phenotype in collected tumors frozen sections were stained using antibody directed against CD206 antigen (Abcam; Ab64693, 1:100, Cambridge, UK). Additional identification of macrophages were performed using an antibody against F4/80 antigen (Abcam; Ab6640, 1:100, Cambridge, UK). To determine the presence of hypoxia-inducible factor 1 in collected tumors, frozen sections were stained using antibody directed against HIF-1 antigen (Abcam; Ab179483, 1:100, Cambridge, UK). To identify the primary antibodies the secondary antibodies conjugated with fluorochromes were used (FITC, Texas Red) (Vector Laboratories, FI-1200, 1:100; Burlingame, USA). Sections were mounted in VECTASHIELD Mounting Medium with DAPI (Vector Laboratories, H-1200). The fluorescence intensity was measured with ImageJ software (NIH). Microscopic observations were performed using an LSM 710 Zeiss confocal microscope (Carl Zeiss Microscopy GmGB, Gottingen, Niemcy).

### Histochemical staining

In days 2 and 7 after DMXAA and digoxin administrations tumors were collected, fixed in PFA and paraffin-embedded. Paraffin sections were examined histochemically (hematoxylin/eosin staining). Analysis of histochemistry specimens were performed using Nikon Eclipse 80i microscope (Nikon Instruments Inc., Melville, NY, USA).

### Determination of immune system cells after antitumor therapy

Mice were sacrificed on the 14^th^ day of the experiment. Tumors were collected for flow cytometric analysis; single-cell suspension was obtained using a digestion mix (0.5 mg/mL collagenase A, Sigma Aldrich; 0.2 mg/mL hyaluronidase type V, Sigma Aldrich; 0.02 mg/mL DNase I, Roche; per 0.25 g of tumor tissue). Red blood cells were lysed using 0.15 M ammonium chloride (Sigma Aldrich). Dead cells were removed by centrifugation using Lympholyte-M gradients (Cedarlane, Ontario, Canada). To identify the subpopulations of T lymphocytes, the following antibodies were used: PE-Cy7^TM^-CD3e, PE-CD4 and APC-CD8a (BD Pharmingen, catalog number: 558431, component: 51-9000790). The titers of antibodies was performed in accordance with the manufacturer’s instructions. Finally, to identify the level of NK cells, an anti-mouse CD49b (pan-NK cells) antibody was used (1 µg/10^6^ cells; eBioscences, catalog number: 17-5971-82). In flow cytometric analyses (BD FACSCanto, BD), gate dividing negative from positive cells was based on isotype antibody control probes: PE-Cy™7 Hamster IgG1_κ_, PE and APC Rat IgG2a_κ_ (BD Pharmingen, catalog number: 558431, component: 51-9000792) or APC Rat IgM (1 µg/10^6^ cells, eBioscences, catalog number: 17-4341-82)^30^. The titers of antibodies was performed in accordance with the manufacturer’s instructions.

### Statistics

For statistical analysis Kruskal-Wallis and multiple comparisons of mean ranks for all groups tests were used. Differences in p values of 0,05 or less were considered significant.
