# Author Correction: Combination of anti-vascular agent - DMXAA and HIF-1α inhibitor - digoxin inhibits the growth of melanoma tumors

**DOI:** 10.1038/s41598-020-62440-x

**Published:** 2020-03-25

**Authors:** Ryszard Smolarczyk, Tomasz Cichoń, Ewelina Pilny, Magdalena Jarosz-Biej, Aleksandra Poczkaj, Natalia Kułach, Stanisław Szala

**Affiliations:** 1 Center for Translational Research and Molecular Biology of Cancer Maria Sklodowska-Curie Institute - Oncology Center, Gliwice Branch, Wybrzeże Armii Krajowej Street 15, 44-101 Gliwice, Poland; 2 Department of Organic Chemistry, Biochemistry and Biotechnology, Silesian University of Technology, Gliwice, Poland; 3 Department of Animal Physiology and Ecotoxicology, University of Silesia, Bankowa 9 St., Katowice, Poland

Correction to: *Scientific Reports* 10.1038/s41598-018-25688-y, published online 09 May 2018

The Acknowledgements section in this Article is incomplete.

“This work was performed within the framework of the project No. UMO-2015/17/N/NZ4/02738. This work was supported by equipment bought for the purposes of the Project: “Silesian BIO-FARMA Center for Biotechnology, Bioengineering, and Bioinformatics” co-financed by European Regional Development Fund within the framework of Innovative Economy Operational Programme 2007–2013.”

should read:

“This work was performed within the framework of the project No. UMO-2015/17/N/NZ4/02738 financed from the funds of National Science Center. This work was supported by equipment bought for the purposes of the Project: “Silesian BIO-FARMA Center for Biotechnology, Bioengineering, and Bioinformatics” co-financed by European Regional Development Fund within the framework of Innovative Economy Operational Programme 2007–2013.”

